# Moyamoya Syndrome (MMS) in a Patient With Sickle Cell Disease (SCD) and Protein S Deficiency

**DOI:** 10.7759/cureus.34314

**Published:** 2023-01-28

**Authors:** Parima Saxena, Hussam Alkaissi, Riddhi Chauhan, John Muthu

**Affiliations:** 1 Internal Medicine, State University of New York Downstate Medical Center, Brooklyn, USA; 2 Internal Medicine, Kings County Hospital Center, Brooklyn, USA; 3 Internal Medicine, Veterans Affairs Medical Center, Brooklyn, USA; 4 Internal Medicine, State University of New York Downstate Health Sciences University, Brooklyn, USA; 5 Kings County Hospital Center, Medicine, Sickle Cell Division, Brooklyn, USA

**Keywords:** endovascular surgery, hereditary protein s deficiency, secondary stroke prevention, moyamoya disease (mmd), sickle cell disease: scd

## Abstract

The association between Moyamoya syndrome (MMS) and sickle cell disease (SCD) has been well-established in pediatric populations; however, limited literature exists documenting the characteristics and management of MMS in adult SCD patients. Studies have indicated the role of endovascular management in secondary stroke prevention for pediatric populations, with no current guidelines available for adult populations. Here, we describe a unique case of MMS in a 30-year-old patient with SCD and incidental protein S deficiency. Our unique case highlights a patient at high risk for neurosurgical intervention due to her hypercoagulable state who has benefitted from medical management. We also discuss current literature for the prevention of secondary cerebral vascular events and the role of further studies involving adult populations with MMS and SCD.

## Introduction

Sickle cell disease (SCD) is caused by a mutation in the beta globin chain of hemoglobin (HbS). It results in inadequate hemoglobin folding during periods of physiological stress, such as hypoxia, hyperosmolar states, infection, and acidosis. Inappropriate hemoglobin polymerization facilitates a sickled morphology, which causes vaso-occlusion, hemolytic anemia, ischemia-reperfusion injury, hyper-coagulability, and widespread inflammation [1]. The degree of vascular injury and inflammation in SCD predisposes patients to a higher rate of cerebral vascular accidents (CVA). Patients with homozygous SCD (HbSS) have an age-adjusted incidence of CVA of 0.61 per 100-person years [2]. CVAs in SCD patients have a multitude of arterial and venous etiologies, including ischemic, hemorrhagic, silent infarcts (asymptomatic infarcts noted incidentally on imaging), dural vein thrombosis and from Moyamoya syndrome (MMS) [3]. Moyamoya vasculopathy is a cerebrovascular condition described as a narrowing of the large intracranial arteries initially, triggering the development of small-vessel collaterals in the later stages of the disease. Reduced blood flow in the internal carotid arteries (ICAs) and their branches predisposes patients to recurrent strokes and transient ischemic events [4]. MMS is defined by a characteristic vasculopathy pattern on imaging alongside epidemiological risk factors such as SCD, neurofibromatosis type I, craniopharyngioma, medulloblastoma, Down syndrome, Graves disease, and renal artery stenosis [5]. Diagnosis is based on vessel narrowing and collateral formation, as seen on magnetic resonance angiography (MRA) [5]. Here, we describe a unique case of MMS in an adult patient with SCD and incidental protein S deficiency. Her case outlines the considerations for stroke diagnosis in adult SCD patients and managing patients with concurrent SCD and hypercoagulable state. 

## Case presentation

**Figure 1:**
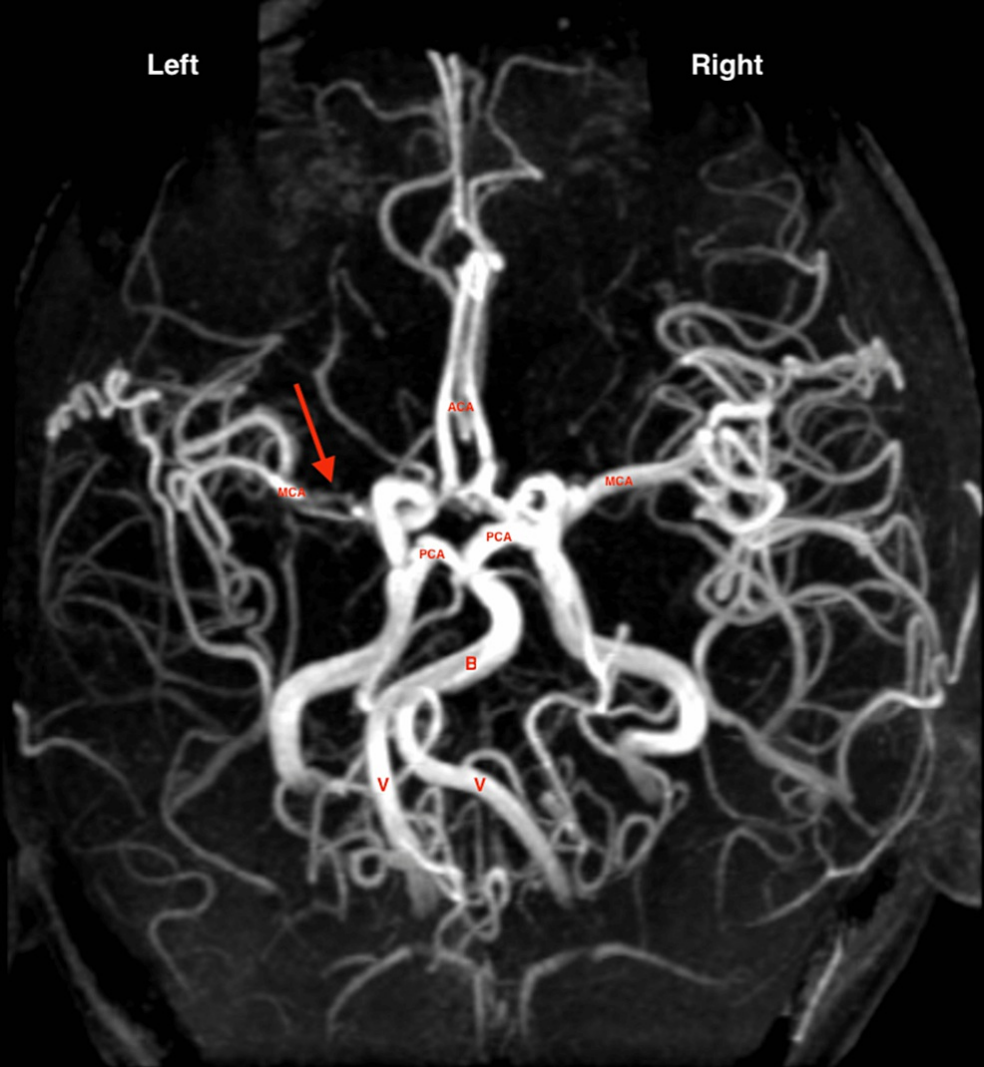
MRA shows a signal void within the left MCA followed by a normal signal in the distal end of segment M1 of MCA and normal M2 segment (left and right panels, shown by arrow). ICA is seen within normal limits with no evidence of stenosis. Significant collateral circulation is seen bilaterally. ACA, anterior cerebral artery; BA, basilar artery; ICA, internal carotid artery; MCA, middle cerebral artery; M1, first segment of MCA; M2, second segment of MCA; M3, third segment of MCA; MRA, magnetic resonance angiography; PCA, posterior cerebral artery; VA, vertebral artery

**Figure 2:**
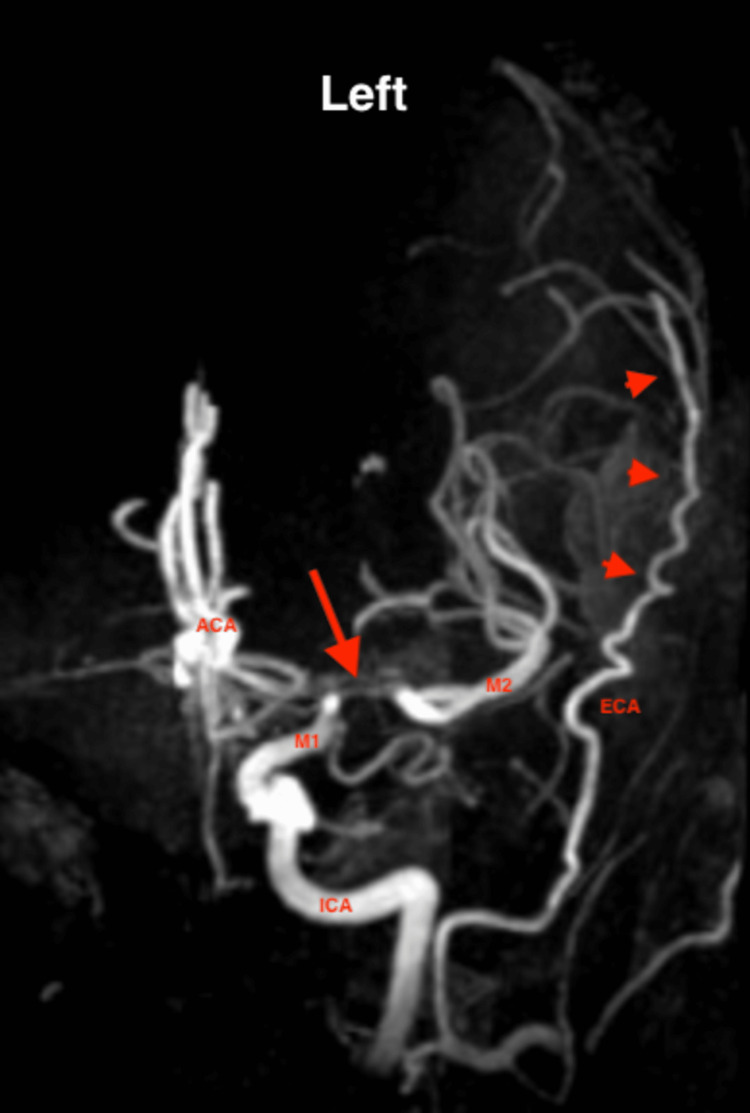
MRA of the left side circulation, focusing on the signal void within the MCA (arrow), and showing the extensive tortuous collateral circulation from the ECA (arrowheads). ACA, anterior cerebral artery; ECA, external carotid artery; ICA, internal carotid artery; MCA, middle cerebral artery; M1, first segment of MCA; M3, third segment of MCA

A 30-year-old female with a past medical history of homozygous SCD and prior pulmonary embolism presented to the sickle cell clinic to establish care. Her SCD (HbSS) is well-controlled, and she reports having rare pain crises and had one prior blood transfusion during her pregnancy a year before the presentation. Her hemoglobin baseline is between 8 and 9 g/dL. She experienced pulmonary embolism during her pregnancy one year before presentation, at 26 weeks of gestation. At that time, she was managed with six months of daily treatment doses of enoxaparin therapy. She has an etonogestrel implant for pregnancy prevention. She was offered hydroxyurea by her prior physician but declined. One month before the presentation, she experienced sudden onset neurological symptoms, where she had new onset slurring of speech and word-finding difficulty, lasting a few hours. MRI brain was performed in an outside facility, for which the patient brought in the report, demonstrating gliotic changes in the left frontoparietal white matter, acute to subacute ischemic changes in the left parietooccipital lobe, and subacute vs. chronic hemorrhagic infarcts in the left parietal lobe; suggesting an extension of prior stroke. Since her symptom onset one month ago, she has been experiencing constant left-sided throbbing headaches unrelieved by codeine. The speech slurring and word-finding difficulty had subsided. She had no vision changes, weakness, numbness, or tingling. The physical exam was unremarkable. Given her concerning symptoms and abnormal MRI report from Guyana, the patient was referred to neurology for further evaluation of stroke in the setting of SCD. The patient was seen in the general neurology clinic, with concern for her neurologic presentation secondary to ischemic stroke with possible hemorrhagic conversion. Given her history of SCD, there also was a concern for arterial embolic stroke or cortical vein thrombosis. As such, the neurology team recommended a complete stroke workup. The brain MRI demonstrated small strokes in the left frontoparietal deep border zone with areas of hemorrosidden deposition, suggesting old hemorrhagic conversion. Magnetic resonance venography (MRV) showed no signs of dural sinus thrombosis. MRA was notable for left ICA terminus stenosis extending into the proximal left anterior cerebral artery (ACA) and middle cerebral arteries (MCAs) (Figures 1-2), consistent with Moyamoya vasculopathy.

Trans-thoracic echo was notable for pulmonary artery systolic pressure of 29, with normal ejection fraction and no structural wall or valvular abnormalities. Given the history of pulmonary embolism, a thrombophilia workup demonstrated low protein S activity of 44% (normal range 57%-123%), normal protein C activity, normal antithrombin C activity, negative cardiolipin antibodies, and negative beta-2 glycoprotein. The coagulation workup was done while the patient was off anticoagulation for a year. She was diagnosed with protein S deficiency and advised to remove the etonogestrel implant to minimize the risk of thrombosis. In addition, she was started on statin and clopidogrel for secondary stroke prevention. The patient was subsequently lost to follow-up for 14 months. She presented again 14 months later to the emergency department with chest pain, at which time she was also 19 weeks pregnant. She was admitted for suspicion of new-onset pulmonary embolism. CT angiography was negative for pulmonary emboli. Chest pain was determined to be of musculoskeletal etiology. She was subsequently discharged with 1.5 mg/kg enoxaparin daily to prevent venous thrombotic events, given concern for combined hyperestrogenic state and protein S deficiency in pregnancy. She was also started on prophylactic transfusion therapy to maintain hematocrit above 30%, to reduce the risk of fetal loss and hypoxia, and for secondary stroke prevention during pregnancy. She successfully delivered via C-section at 37 weeks gestation with no complications. She has been followed for three years since her initial diagnosis of MMS and protein S deficiency. She is currently following up with the sickle cell and neurology clinic with an observational approach to managing her MMS and protein S deficiency. She has not had any further neurological symptoms or signs of thrombotic events. The current medication regimen includes aspirin 81 mg and enoxaparin 1 mg/kg.

## Discussion

Cerebrovascular accidents are a common complication in patients with SCD, with the pathogenesis of silent infarcts appearing early in life. Current literature suggests that attachment of sickled cells to vascular endothelium leads to a cascade of endothelial activation and vascular damage, as implicated in vaso-occlusion, intimal hyperplasia, fibrosis, and thrombosis [3]. MMS in SCD patients likely stems from vascular damage leading to progressive stenosis of the ICAs, resulting in the formation of fragile arterial collaterals that are easily subjected to damage and impairment of blood flow during periods of vaso-occlusion [3]. MMS can be found in 20%-35% of pediatric patients with SCD who undergo cerebral angiography, and if present, indicates severe cerebrovascular disease with a high risk of recurrent CVAs [3, 5]. To date, most studies in MMS and SCD have involved pediatric populations. One prominent study by Dobson et al. involved retrospective analysis of 44 pediatric SCD patients with CVA and evaluated recurrent stroke risk in these patients, followed for an average of 8.1 years after initial CVA. 19/44 of their patients demonstrated findings consistent with MMS on MRA. Their analysis demonstrated that patients with MMS were significantly more likely to have a second CVA than SCD patients without MMS (57.9% vs. 28%, p < 0.05). They also found that children with MMS had a median time to recurrent stroke of 3.1 years. They could not estimate the time to reoccurrence in patients without MMS due to the low likelihood of recurrent CVA during their study period. Their results indicated that MMS increased recurrent CVA risk with a hazard ratio of 2.40 (confidence interval, CI = 0.85-6.75) [6]. The authors also noted that MMS in SCD led to more significant intellectual impairments and cognitive deficits than in patients without MMS [6]. Similarly, Soares et al. documented a case of an eight-year-old boy with SCD and MMS who developed recurrent stroke two years after initial CVA after noting symptoms of inattentiveness in the classroom [5]. The authors of this report suggest that there may be a role of endovascular therapy in the prevention of future CVAs [5]. Management of MMS in SCD patients can include conservative (medical) or surgical management. Endovascular surgery has shown an important role in reducing the risk of recurrent CVA in pediatric populations through a procedure known as encephaloduroarteriosynangiosis [7]. Medical management usually involves chronic transfusions to decrease the number of sickled RBCs, with or without hydroxyurea [8]. Yang et al. conducted a retrospective study involving 15 pediatric patients with MMS and SCD. Seven patients were surgically managed; eight were medically managed and followed up for 11.6 years. They noted that four patients in the medical management arm of the study had recurrent CVAs, whereas none of the revascularization patients experienced recurrent CVAs [8].

Similarly, a recent meta-analysis by Agulair-Salinas et al., which pooled 53 patients from seven studies who underwent revascularization procedures, found that the rate of ischemic stroke-free survival was 94.3% (95% CI 83.3-98.1) and the incidence rate of ischemic stroke was 1.42 events/100 patient-years (95% CI 0.46-4.4). Likewise, a larger systematic analysis by Terell et al. reviewed the data from 13 studies of pediatric populations. Early detection and surgical intervention significantly improved stroke recurrence and neurocognitive outcomes [9]. All of these studies were conducted using pediatric populations and are retrospective. These studies did not consider the role of chronic transfusions before surgical intervention. Given that these studies are based on pediatric populations, the role of endovascular intervention in adults remains unknown. 

Chronic transfusion therapy remains the gold standard for managing secondary stroke prevention for MMS-SCD patients. Transfusions can be performed every 3-6 weeks to maintain HbS levels <30%, with overall hemoglobin levels between 9 and 12.5 g/dL. The SWITCH trial, a phase 3 multicenter trial, evaluated the role of transitioning from chronic transfusion therapy to hydroxyurea and noted that transitioning to hydroxyurea was associated with increased stroke risk in pediatric patients [10]. Current American Society of Hematology (ASH) guidelines (2020) recommend that there is insufficient evidence for or against neurosurgical intervention in pediatric and adult patients and that candidates should undergo multi-disciplinary evaluation before revascularization [11]. 

Only a handful of cases are documented in the literature describing MMS in adult SCD patients [12-15], with most cases and management regimens focused on pediatric populations. Of the cases in adults, there has been no mention of the role of surgical management, and current case reports describe medical management [12-14].

## Conclusions

Here, we present a case of MMS in a patient with SCD patient with concomitant protein S deficiency. Our unique case highlights a patient at high risk for neurosurgical intervention due to a concomitant hypercoagulable state. Thus far, our patient has benefitted from medical management for secondary stroke prevention and yearly monitoring for MMS progression. Future studies should consider a retrospective analysis of patients diagnosed with MMS in adulthood to determine outcomes of medical vs. revascularization and consider the surgical risks associated with neurosurgical intervention in adult populations. Overall, we outline a unique case of MMS-SCD in an adult patient with protein S deficiency and consider the role of conservative/medical management of MMS in adult patients. 
